# Assessment of Heart Rate Recovery with GATED-Myocardial Perfusion Scintigraphy Outcome in Patients with Coronary Artery Disease: A Retrospective Study and Institutional Experience

**DOI:** 10.4274/mirt.60252

**Published:** 2016-09-29

**Authors:** Yusuf Ziya Tan, Semra Özdemir, Burak Altun, Fatmanur Çelik

**Affiliations:** 1 Onsekiz Mart University Faculty of Medicine, Department of Nuclear Medicine, Çanakkale, Turkey; 2 Onsekiz Mart University Faculty of Medicine, Department of Cardiology, Çanakkale, Turkey

**Keywords:** coronary artery disease, heart rate recovery, stress myocardial perfusion scintigraphy

## Abstract

**Objective:**

This study aimed to investigate the effects of assessment with myocardial perfusion scintigraphy (MPS) and heart rate recovery (HRrec) measurements in combination to evaluate the current status of patients with a diagnosis or suspicion of coronary artery disease (CAD).

**Methods:**

A total of 350 patients were included in the study. CAD group consisted of 200 patients with stable angina pectoris and a known history of CAD, while the control group consisted of 150 patients with suspicious stress test who had no history of known CAD. In order to calculate the HRrec index, the treadmill exercise test was performed in all patients according to the Bruce protocol. The MPS results were evaluated for the presence or absence of myocardial ischemia and infarction by visual and quantitative (summed stress score and summed difference score) assessments.

**Results:**

When the MPS results and HRrec were evaluated together, there was no statistically significant difference in the non-CAD group. But, when GATED-MPS was evaluated alone in the triple-vessel patient group, 27 (36%) patients were found to be normal while evaluated with HRrec, four (5.3%) patients were found to be normal.

**Conclusion:**

HRrec measurements obtained during stress MPS is important in patient evaluation. Therefore, evaluation of MPS results and HRrec measurements together may provide a more accurate estimation of possible presence of CAD in patients.

## Abstract (tr)

**Amaç:**

Bu çalışmada koroner arter hastalığı (KAH) olan ya da şüphesiyle incelenen hastaların mevcut durumlarının daha iyi değerlendirilmesi için miyokard perfüzyon sintigrafisi (MPS) ve kalp toparlanma hızlarının birlikte değerlendirilmesinin sonuçlar üzerine olan etkisi araştırıldı.

**Yöntem:**

Çalışma retrospektif özellikte olup toplam üç yüz elli hasta dahil edildi. Anjina pektoris ve KAH öyküsü bulunan 200 kişi hasta grubunu, KAH öyküsü bulunmayan 150 hasta ise kontrol grubunu oluşturdu. Hastalara Bruce protokolüne göre efor testleri yaptırılarak kalp toparlanma hızları ölçüldü. MPS sonuçları kalp hızı toparlanma indeksleri ile birlikte değerlendirildi.

**Bulgular:**

Koroner hastalığı olmayan hasta grubunda MPS sonuçları ve kalp hızı toparlanma indeksleri birlikte değerlendirildiğinde istatiksel olarak anlamlı fark bulunmadı. Ancak, üç damar hasta grubunun değerlendirilmesinde sadece MPS sonuçları yapılan değerlendirmede hastaların 27’si (%36) normal bulunurken, kalp hızı toparlanma indeksleri ile birlikte değerlendirildiğinde hastaların dördü (%5,3) normal olarak saptandı.

**Sonuç:**

Stres MPS sırasında elde edilen kalp hızı toparlanma ölçümleri hastaların değerlendirilmesinde önemlidir. Bu nedenle, MPS sonuçlarının kalp hızı toparlanma ölçümleri ile birlikte değerlendirilmesi olası KAH varlığını daha doğru öngörebilir.

## INTRODUCTION

Coronary artery disease (CAD) is one of the leading causes of mortality in developed countries.

Due to the incidence of CAD, there is a need for accurate, inexpensive, and non-invasive imaging methods for both diagnosis and monitoring. Exercise tests were the initial tests employed in diagnosis of CAD.

Changes in heart rate during and immediately after exercise determine the balance between the sympathetic system and vagal activity. During the recovery period after exercise, as the sympathetic activity that increased during exercise reduces, parasympathetic activity increases and causes a reduction in heart rate (1).

Heart rate recovery (HRrec) refers to the decrease in heart rate after exercise. HRrec index is calculated by subtracting the heart rate at the 1^st^, 2^nd^ and 3^rd^ minutes of the recovery period from the maximum heart rate in a patient performing a submaximal or maximal stress test (2).

HRrec index is an important marker of vagal activity, and there are many studies showing that it is a strong predictor of deaths due to all causes as well as cardiovascular reasons (3,4,5).

Many studies have shown that the HRrec index in the 1^st^ and 2^nd^ minutes strongly predicts prognosis in coronary artery patients and reported that those with low HRrec have a significantly higher risk of mortality (5).

GATED-myocardial perfusion scintigrapy (MPS) is a nuclear medicine method that is currently being used widely for the diagnosis and monitoring of CAD (6).

GATED-MPS uses a radioactive marker such as ^99m^Tc-methoxyisobutylisonitrile or Tl201 in patients undergoing treadmill or pharmacological stress. Data obtained under stress and rest conditions using MPS-single photon emission computed tomography (SPECT) and GATED [simultaneous with electrocardiography (ECG)] methods is used to investigate myocardial perfusion in the left ventricle and heart wall movements on segmentary analyses on polar graphics prepared on slices in transaxial, coronal and sagittal planes.

MPS results can be assessed both visually and quantitatively. Abnormal perfusion images may be observed in balanced ischaemia cases with triple vessel disease and also in cases with insufficient stress test (7).

Both exercise and GATED-MPS provide false positive and false negative results if used alone for the assessment of CAD. Therefore, in this study we aimed to investigate the effects of assessment with MPS and HRrec measurements in combination to evaluate the current status of patients with a diagnosis or suspicion of CAD.

## MATERIALS AND METHODS

### Study Population

The study was retrospectively planned on patients who applied to Çanakkale University Faculty of Medicine, Nuclear Medicine Department for MPS with the aim of investigating CAD. The study included 350 patients comprising 225 women and 125 men. The CAD group consisted of 200 patients with known history of CAD and stable angina pectoris, while the control group included 150 patients with no known history of CAD and who had a suspicious stress test.

Patients were excluded from the study if they had congestive heart failure, advanced degree of aortic stenosis, severe hypertrophic cardiomyopathy, malignant hypertension, uncontrolled rhythm disorders, acute ischemia, chest pain within the previous two days, if they could not perform stress test due to orthopedic problems, those with musculo-skeletal problems, peripheral artery disease, psychiatric problems, those using medications affecting the autonomic system, and those under the age of 20 and above the age of 65 years.

The study was completed after receiving permission from the local ethics committee.

Heart rate and recovery index measurements;

### Basal heart rate:

All patients had a basal ECG performed in a quiet room at body temperature, with the patient awake and resting, before the study to measure basal heart rate (HRrest).

### Maximum heart rate (HRmax):

The HRmax is the highest heart rate obtained by exercise linked to age. HRmax was measured with the aid of the Fox et al. (8) formula [HRmax: 220-(age)].

### Target heart rate (THR) measurement:

THR was calculated using the Karvonen (9) method. Maximal effort performance THR was accepted as a test reaching 85% and above.

### Treadmill stress test and HRrec index measurements:

 A treadmill stress test was performed in accordance with the Bruce protocol prior to GATED-MPS in order to increase myocardial perfusion in all patients. The stress test was stopped when 85% of the calculated maximal heart rate according to age was reached. To calculate the HRrec index, when maximal heart rate was reached, the heart rates at the 1^st^, 2^nd^ and 3^rd^ minutes of the recovery period before beginning a relaxing walk were subtracted from the maximal heart rate and were recorded as Rec 1, Rec 2 and Rec 3 HRrec index.

### HRrec index cut-off values:

 In our study, instead of using our own cut-off values for normal and abnormal HRrec, we benefitted from the cut-off values obtained in previous studies. The HRR was accepted abnormal if < or =12 beats/min during the first minute after exercise (10).

### GATED-MPS:

 Patients underwent stress-rest protocols in a single day. All patients discontinued cardiac glycosides one week prior to the test while other antihypertensive medications were stopped 48 hours before the procedure. Stress images were taken 45 minutes after 10 mCi ^9^9mTc-sestamibi injection, with resting images obtained 4 hours after initial imaging and 60 minutes after 30 mCi ^99m^Tc sestamibi injection.

GATED-MPS images were taken with a low energy, high resolution, double head gamma camera (GE, Infinia) fitted with a parallel-slit collimator synchronized to ECG from 45° right anterior to 45° left posterior oblique, with 140±20% keV energy peak, 64x64 matrix, in supine position. Each image took 35 seconds and a total of 32 images were obtained.

After processing the raw data obtained from the patients with the aid of a computer, it was then evaluated both quantitatively and visually.

### Statistical Analysis

Research data was uploaded to the SPSS 19.0 statistical program in the electronic environment and the analyses were performed. Statistical evaluation used the chi-square analysis, and p<0.05 was accepted as significant.

## RESULTS

**Figure 1:**
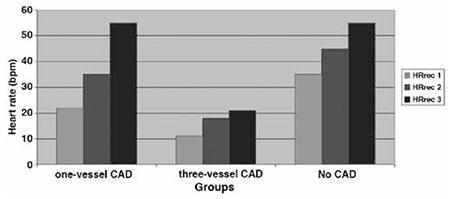
Heart rate recovery index shows that in patients with three-vessel lower than the other groups CAD: Coronary artery disease, HRrec: Heart rate recovery

The clinical characteristics of patients in coronary artery and control groups are presented in Table 1. The mean age in the CAD group was 56±9 years, and was 52±11 years in the control group. The CAD patients comprised 70% female and 30% male, while the control group comprised 56.6% female and 43.3% male. Comparison of the clinical characteristics and cardiac risk factors between the groups did not reveal any statistically significant difference in terms of age distribution. However, it was observed that the number of female cases was greater than the number of male cases. In terms of cardiac risk, there was no significant difference between the CAD group and the control group (p>0.005). In the CAD group 55% had single vessel, 7.5% had double vessel and 37.5% had triple vessel disease (Table 1). While there was no clear difference between the HRrec in the single and double vessel disease cases in the CAD group, the HRrec in the triple vessel disease group was clearly lower than the other two sub-groups (Figure 1). When the HRrec values are compared in the patient and control groups, the HRrec in the 1^st^ and 2^nd^ minute in the CAD group was identified to be significantly lower than the control group (p=0.002, Table 2).

The GATED-MPS results of all participants are presented in Table 3. In the CAD group, 47/200 (23.5%) of patients were normal and 153/200 (76.5%) had ischemia; while in the control group, 128/150 (85.3%) were normal and 22/150 (14.6%) were found to have ischemia (p<0.001, Table 3).

When the MPS results are evaluated together with the HRrec, there was no statistical difference observed in the control group. When evaluated with HRrec in the CAD group in single vessel patients 9% had ischemia of the 21.8% of patients found to be normal, while there was no difference for double vessel patients. A patient with three-vessel disease was presented Figure 2A, 2B. When GATED-MPS is evaluated alone for the triple vessel patient group, 27 (36%) patients were found to be normal while evaluated with HRrec, four (5.3%) were found to be normal (Table 4).

## DISCUSSION

The results of this study, similar to previous studies, indicate that the HRrec index for the first and second minutes was lower in CAD patients as compared to controls. The other significant finding of the study is that the HRrec index correlated with the extent of CAD. These findings indicate that it may be an important parameter to evaluate in patients with CAD. Additionally, the positive contribution of evaluation with HRrec and MPS in combination was shown with the reduced false negative results.

Heart rate is stated as the number of heart beats per unit time. Heart rate changes in many situations depending on the body’s needs, oxygen and carbon dioxide levels in the blood, physical and mental activity, etc. (11). There are many studies showing that a low resting heart rate reduces mortality. In healthy and asymptomatic individuals, the heart rate falls rapidly within the first 30 seconds after exercise followed by a slower reduction (12).

There are studies showing that heart rate is disrupted in uncomplicated heart diseases even before the development of symptomatic CAD (13).

Cole et al. (14) showed that the lack of expected decrease in heart rate in the 1st minute after exercise (≥12 beats/min) is a marker of reduced vagal activity. This situation may be a strong marker for general mortality independent of heart rate changes due to work load, the presence of myocardial perfusion defect, and during exercise.

A study by Lima et al. (15) reported that there was no dependent relationship between abnormal HRrec and summed different score. In contrast, Georgoulias et al. (16) showed that abnormal HRrec index was an important indicator of ischemia identified on MPS-SPECT images.

Inconsistency between the results of MPS and coronary angiography (CAG) is frequently encountered. The lack of clear coronary artery stenosis on CAG of patients with ischemic perfusion findings is thought to be due to false positive results of scintigraphy. In circumstances that affect the diagnostic accuracy of MPS, such as insufficient stress administration and triple vessel disease, the sensitivity decreases to 60% (17,18,19).

Vivekananthan et al. (20) reported that angiographic severity of CAD, left ventricle function, exercise capacity and HRrec index may be independent predictors of mortality.

HRr is also affected by other factors such as age, heart failure, previous myocardial infarction (MI), diabetes, hypertension and smoking.

In our study, we encountered false positive results in 17 patients (11.3%) with normal CAG. There was no ECG or echocardiography finding to explain the false positivity of MPS. We encountered false positive results in 19.2% of those with single vessel disease on CAG, and in 42.6% of those with multiple vessel disease. The main aim of our study was to investigate how could the current situation of patients with a diagnosis or suspicion of CAD be evaluated more accurately. As a result, when we evaluated the HRrec and MPS results together for all participants, we found that accuracy was higher than that obtained from HRrec or MPS alone. We believe that the false negative evaluations on MPS results, similar to those with triple vessel disease, may be reduced with this approach.

### Study Limitations

The first limitation of the study is that the stress test before MPS was completed according to the Bruce protocol. Patients reaching maximum heart rate on the treadmill continued with a slow walking pace for up to a minute after the end of stress. As a result, the HRrec obtained from the 2^nd^ minute were evaluated as the cooling period. Second, in the CAD group, patients were not assessed for MI and coronary artery bypass graft (CABG). There is a need for studies that take MI and CABG durations into account. Finally, our study is a retrospective study conducted on a small group of patients, this issue should be studied in larger groups in the future.

## CONCLUSION

Evaluation of HRrec measurements obtained during GATED-MPS provided a positive contribution and may be used to estimate the current situation of CAD patients more accurately.

## Ethics

Ethics Committee Approval: The study was approved by the Çanakkale Onsekiz Mart University Local Ethics Committee, Informed Consent: Consent form was filled out by all participants.

Peer-review: External and internal peer-reviewed.

Financial Disclosure: The authors declared that this study has received no financial support.

## Figures and Tables

**Table 1:**
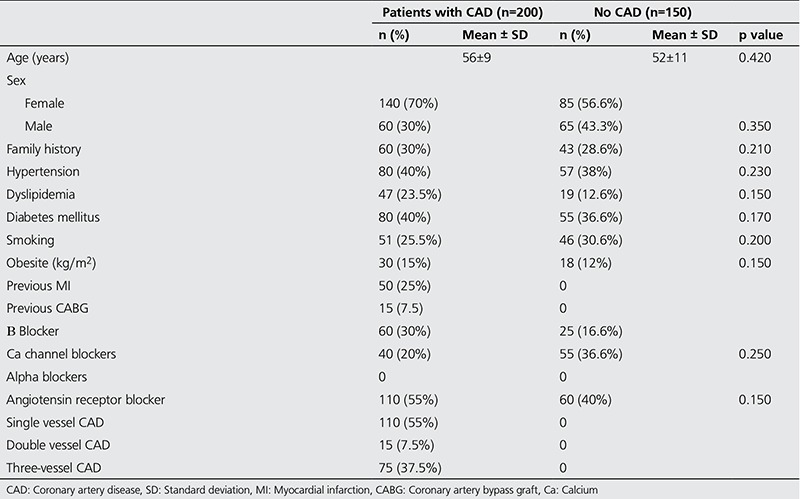
Baseline patient characteristics of the coronary artery disease and the control groups

**Table 2:**
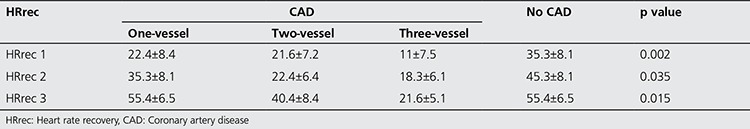
Heart rate recovery indices of the groups

**Table 3:**
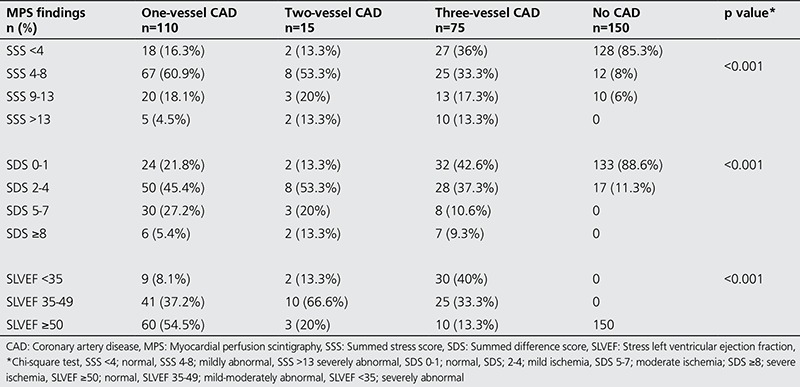
Myocardial perfusion imaging findings in the coronary artery disease and control groups

**Table 4:**
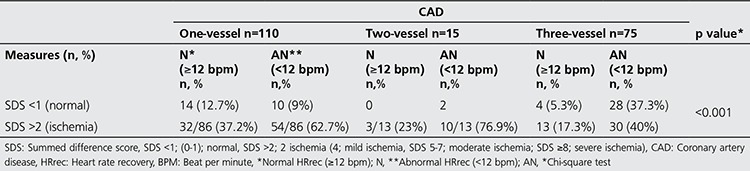
Comparison of myocardial perfusion imaging findings and heart rate recovery with coronary artery disease

**Figure 2A:**
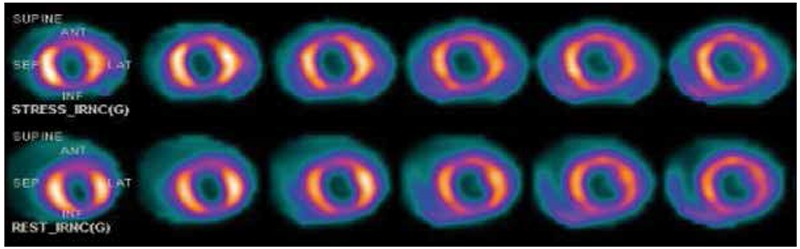
The view of a 50-year-old male patient with myocardial perfusion scintigraphy. Myocardial perfusion scintigraphy in all areas are monitored reduced and heterogeneous uptake. Three-vessel disease was detected in the patient’s coronary angiography

**Figure 2B:**
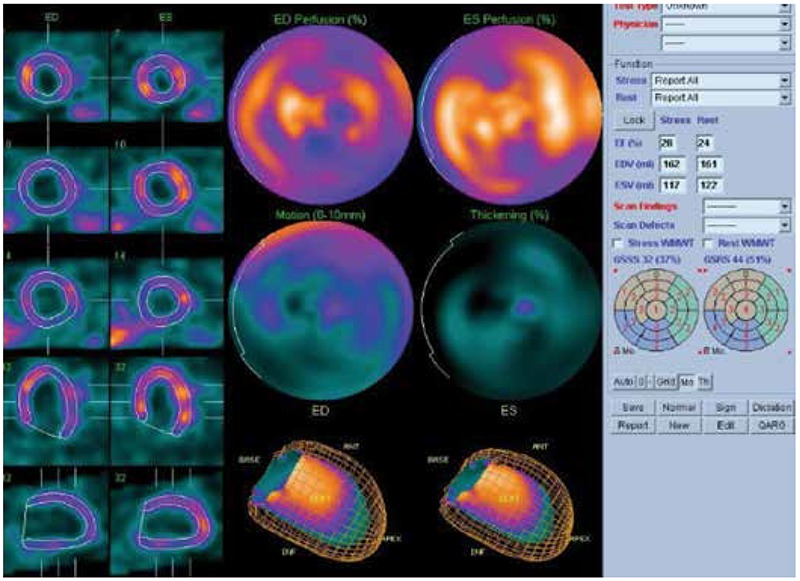
Quantitative gated single photon emission computed tomography findings of the same patient. In the GATED-single photon emission computed tomography imaging were found stress ejection fraction: 31%, resting ejection fraction: 24%
